# A Dynamic Approach to Economic Priority Setting to Invest in Youth Mental Health and Guide Local Implementation: Economic Protocol for Eight System Dynamics Policy Models

**DOI:** 10.3389/fpsyt.2022.835201

**Published:** 2022-04-29

**Authors:** Kenny D. Lawson, Jo-An Occhipinti, Louise Freebairn, Adam Skinner, Yun Ju C. Song, Grace Yeeun Lee, Sam Huntley, Ian B. Hickie

**Affiliations:** ^1^Faculty of Medicine and Health, Brain and Mind Centre, University of Sydney, Sydney, NSW, Australia; ^2^Computer Simulation & Advanced Research Technologies (CSART), Sydney, NSW, Australia; ^3^Research School of Population Health, Australian National University, Canberra, ACT, Australia

**Keywords:** economics, mental health, priority setting, system dynamics, policy

## Abstract

**Background:**

Mental illness costs the world economy over US2.5 Bn each year, including premature mortality, morbidity, and productivity losses. Multisector approaches are required to address the systemic drivers of mental health and ensure adequate service provision. There is an important role for economics to support priority setting, identify best value investments and inform optimal implementation. Mental health can be defined as a complex dynamic system where decision makers are challenged to prospectively manage the system over time. This protocol describes the approach to equip eight system dynamics (SD) models across Australia to support priority setting and guide portfolio investment decisions, tailored to local implementation context.

**Methods:**

As part of a multidisciplinary team, three interlinked protocols are developed; (i) the participatory process to codesign the models with local stakeholders and identify interventions for implementation, (ii) the technical protocol to develop the SD models to simulate the dynamics of the local population, drivers of mental health, the service system and clinical outcomes, and (iii) the economic protocol to detail how the SD models will be equipped to undertake a suite of economic analysis, incorporating health and societal perspectives. Models will estimate the cost of mental illness, inclusive of service costs (health and other sectors, where necessary), quality-adjusted life years (QALYs) lost, productivity costs and carer costs. To assess the value of investing (disinvesting) in interventions, economic analysis will include return-on-investment, cost-utility, cost benefit, and budget impact to inform affordability. Economic metrics are expected to be dynamic, conditional upon changing population demographics, service system capacities and the mix of interventions when synergetic or antagonistic interactions. To support priority setting, a portfolio approach will identify best value combinations of interventions, relative to a defined budget(s). User friendly dashboards will guide decision makers to use the SD models to inform resource allocation and generate business cases for funding.

**Discussion:**

Equipping SD models to undertake economic analysis is intended to support local priority setting and help optimise implementation regarding the best value mix of investments, timing and scale. The objectives are to improve allocative efficiency, increase mental health and economic productivity.

## Introduction

### The Health and Economic Impact of Mental Illness

Mental health is increasingly a policy priority (1). Mental illness is estimated to cost the world economy US$2.5 trillion each year (2), the leading cause of mortality in 14–45 year olds (3, 4) and, in Australia, 15% of 15–24 experience high or very high levels of psychological distress (5). The associated health care costs and productivity losses amount to $40–70 bn in Australia (6). Modifiable drivers of mental health include interdependent structural and individual factors, such as environment, education, employment, health and social services, and individual behaviours (7). Overall, mental health is a systemic challenge that extends beyond the health sector. This requires a systematic policy response with a focus on prevention, reconceptualising mental health as an asset and where improvements in mental capital (cognitive, emotional, and wellbeing) can lead to improved population health, and social and economic prosperity (8).

### Policy Challenge: Investing Explicitly in Multi-Sector Approaches

It is increasingly recognised that a whole-of-government approach is required to develop and coordinate multi-sector responses, tailored to local implementation conditions (5–7). Further, there is a policy direction towards place-based and outcome-based budgeting to overcome siloed approaches, incentivise multisector coordination and encourage joined-up and comprehensive person-centred care (5, 9). Such aspirations meet with significant operational challenges when mental health has traditionally been the domain of health sector and may be significantly underfunded (10). From a systems perspective, the flow-on impacts from investing in mental health to wider intersectoral benefits should be considered in funding and implementation decisions. However, while there is a need for a systems approach to investment there are significant gaps in evidence regarding whether services are efficiently delivered, effective or cost effective (11).

### An Opportunity for Economics to Support Priority Setting and Investment Decisions

There is an opportunity for economics to help generate and translate evidence to support investments in mental health, with wider societal impacts. From an economics lens, given scarce or finite resources (e.g., skilled people, time, and physical locations) there is a need to prioritise and allocate funding between different spending areas (health and non-health) and particular interventions (policies, programs, technologies). To improve population mental health outcomes overall, it is important to understand both the investment required, cost and benefits from implementation, the budget available, and to undertake an explicit option appraisal process to identify best buys.

Evidence of impact is one consideration for decision-makers alongside other concerns, including preferences, ethics, and practical operational constraints such as contractual obligations. To support the process of priority setting, there are longstanding approaches such as Program Budgeting Marginal Analysis (PBMA) and Multi-criteria decision analysis (MCDA) (12–15). These processes draw together multiple stakeholders and identify intervention candidates for investment (and disinvestment) incorporating economic evaluation evidence alongside wider considerations, if relevant.

### Complex Systems, Priority Setting, and Challenges to Traditional Economic Modelling

As part the priority setting process, economic evaluation is one key input. Given the full impacts of interventions can take time to manifest, economic modelling is necessary to project costs and outcomes beyond evaluation studies. However, traditional modelling approaches may not be ideally suited when the challenge is to support decision makers to manage complex systems and implement priorities prospectively.

In health economic modelling, for example, the dominant modelling approaches were purposed for health technology assessment (HTA) and relatively simple interventions, such as pharmaceuticals and surgical technology (16–18). Disease transmission models (e.g., Decision trees and Markov models) generate risk-to-event equations, observed trial effect sizes adjust transmission rates, and the focus is on “final” or cumulative outcomes and costs over the lifetime of a patient cohort, such as Quality-Adjusted Life years (QALYs). Notably, the implementation context is rarely explicitly modelled, but rather embedded within risk-to-event equations. The potential limitation is that evaluation results are implicitly assumed to hold across time and between contexts. Further, results are then scaled to the population eligible for a particular intervention rather than modelling the population demand explicitly, which can vary over time and with implications for service capacities to meet needs. To identify best-buys, it is common for league tables’ to be generated that ordinally rank individual interventions in order of cost effectiveness. For combinations of interventions, there is a need to impose exogenous assumptions regarding potential additive or multiplicative effects. Overall, these methods have been influential in HTA where implementation context can be relatively simple and static, where one product can be swapped for another in a well-resourced supply chain. However, such approaches may not be ideally purposed for the challenge of priority setting and managing dynamic service systems and where local implementation context is vitally important.

Human services and public health, including mental health, may be described as complex systems (19–23). There is an interdependence of demand and supply of different services that influence outcomes over time and summative outcomes. On the demand side, there are changing populations and multiple causal drivers, and on the supply side there are often coupled service systems (e.g., GP screening and referral to allied health providers) with capacity constraints, queuing and delays, and the onset of positive or negative feedback effects. The impact from implementing particular interventions on cumulative outcomes is neither independent from other interventions and local context, nor static. Rather, the features of complexity can result in population level outcomes that are dynamic, non-linear and emergent over time (19–22).

The practical challenge is to support decision makers to manage dynamic systems prospectively. Historical estimates of cost effectiveness may not hold going forward, but are conditional upon local context, subject to variation over time, and where cumulative positive impacts are dependent on the presence of complimentary interventions (19). To optimise implementation and improve allocative efficiency, it is important to support decision makers to actively manage the system as a whole.

### Dynamic Simulation Modelling and Mental Health

With the latest advances in computing and data analytics, complex systems can be simulated offering an opportunity to advance decision support. There is now an extensive literature on dynamic simulation modelling (DSM), guidance documents regarding the process to select and build different model types, including participatory approaches to co-design models with local stakeholders, and an array of empirical examples (24–27).

There are various SD models built to improve mental health outcomes which have demonstrated the presence and significance of complexity (19–22). The key advance is modelling the system and implementation conditions explicitly and using mathematical approaches to capture dynamic behaviours. Such models have provided key insights regarding the potential impacts of an array of social and service level interventions, including the synergetic or antagonist effects of interventions to better generate positive population outcomes. DSM has also been important to demonstrate potentially counter-intuitive and unintended harms from introducing interventions without considering implementation context. For example, public awareness campaigns for mental health and screening for psychological distress may lead to increased self-harm and suicides unless there are also sufficient downstream service capacities (20). A key value of simulation modelling is to learn from the model itself and not only as a tool to project evaluation evidence. DSM have the capability to test out in a simulated environment whether changing the mix, scale and timing of intervention combinations can significantly impact population outcomes over time, before real world implementation (19–22). That is, even if evaluation studies demonstrate cost effectiveness, the impacts may be improved by adjusting complementary services that cumulative impacts depend upon (e.g., better balance of service demand and supply conditions). The models can also identify research priorities where key leverage points are identified but where there is an absence of intervention evidence (19–22).

### Opportunities for Dynamic Approaches to Economic Priority Setting

DSM models have been primarily designed to provide important qualitative insights regarding how a system functions, emergent outcomes, key leverage points and the combinations of interventions with the greatest combined effects (19–22). From an economics perspective, this is necessary but not sufficient to support policy making. A key system constraint is scarce resources and the fundamental decision challenge is optimal allocation. The best combination of investments is also conditional upon the cost of implementation and the budget available. Therefore, there is a need for DSMs intent on guiding decision making that has resource implications to incorporate economic considerations including costs, economic outcomes, budget constraints and embed decision-analytic components that guide an optional appraisal to identify the best-value interventions. This can then also be used to advocate for additional resources and the potential return on investment. There are few examples of economic analysis using DSM beyond infectious disease modelling (28–36).

The challenge for economists is not only to engage with DSM but to advance the associated economic methods, where necessary. For priority setting, there is an opportunity to develop dynamic portfolio approaches to identify the best-value mix of interventions to balance the demand and supply of services (35, 37). Further, there is a need for adaptive decision analysis, where decision-makers and researchers can use the models to manage the system prospectively. It is also important to enable models to take either a societal and multi-sector perspective (including, but beyond QALYs) to support the operationalising of whole-of-government approaches. This also enables a fuller assessment of the societal value of investments to inform allocation of funding to particular sectors. It is also important to understand, where possible, the distribution of costs and benefits across sectors and households to inform a change management process if reallocation of resources between and within sectors is required (38).

### Research Objectives

The purpose of this protocol is to detail the approach to equip eight system dynamics (SD) models across Australia to support priority setting and guide portfolio investment decisions to invest in youth mental health and the population more generally. This work is central to the “Right care, first time, where you live Program.^1^” The intention, as part of a multidisciplinary team, is to develop a macro-level simulation modelling framework, accompanied by a user-friendly dashboard to support local decision makers manage complexity, invest more strategically, and improve mental health and wider economic outcomes.

## Methods

### Study Setting and the Objectives of the System Dynamics Policy Models

This study will develop eight SD models for regional decision makers, over a 4-year period with each model taking approximately 6 months to complete. The eight sites have been selected (two metropolitan, two outer urban, two regional, and two rural/remote sites) to capture variation in socioeconomic conditions, population density, demographic profile, mental health risk profile, and mental health service infrastructure and access (39).

The objectives of the SD model and economic analysis are threefold: (i) to guide strategic priority setting to identify and invest in the best mix of services, relative to a budget(s), and also inform the optimal scale and timing of implementation, (ii) to support the on-going management of the system over time to actively maintain balance in the demand for and supply of services, and (iii) help identify research priorities, such as where key leverage points to improve outcomes are identified but where relevant interventions have not yet been developed. The detail provided in this protocol is intended to be consistent with CHEERS checklist (40).

### Multidisciplinary Process of Building the Model

There are three interrelated components to the overall modelling process, each with separate protocols: (i) a participatory approach (41), (ii) the technical model building (39), and (iii) equipping the model to undertake economic analysis (this paper). The participatory approach is led by research practitioners experienced in systems thinking and complex systems modelling and includes three workshops including diverse stakeholders such as researchers, decision-makers, service planners, and those with lived experience of mental illness. The process provides opportunities for relevant stakeholders to steer the model purpose, structure, and intervention priorities. This helps ensure both face validity of the model logic, assumptions and economic approach and stimulates on-going interest to use and update the model going forward.

The development of the model is led by an experienced system dynamics modeller in collaboration with a multidisciplinary research team with expertise in epidemiology, social science, psychology, biostatistics, health and social policy, and economics. The team works to convert the conceptual model developed by stakeholders into a computational model, iteratively modifying it through engagement with research evidence, available data, and stakeholder feedback. The focus is on creating a macro-level model of the local system, including population, services and health outcomes. Each model will also undergo independent review of its structure and equations to minimise the potential for errors.

The economist will work alongside the system dynamics modeller to integrate into the model the necessary costs, economics outcomes, budgets, and decision frameworks to equip and purpose the model to guide investment decisions. The focus is on priority setting and portfolio optimisation at a macro, or system level, to improve allocative efficiency. Evaluation evidence, where it exists, can be used as an input into the model to project local impacts over time.

The economic approach is embedded within the participatory process and so open to shared decision making to inform the approach. The reflexive nature of the process, and the on-going development and refinement of the model, is the main reason that the methodology section that follows is a relatively high-level description of intended approach, rather than an exhaustive list of all the specific steps, calculations, and data sources. A more detailed Standard Operating Procedure (SOP) is provided (Supplementary file 1) for purposing the model to undertake economic analyses, including examples data sources, calculations, and steps in analysis. In the main body of the paper, the figures provided are unpublished illustrations from an SD model built previously for the North Coast Collective, in New South Wales.^2^ All figures are provided with permission.

### Overview of the Intended System Dynamics Policy Models

#### Model Structure

A full description of the intended modelling approach, scope, parameters, and data inputs is provided elsewhere (39). To contextualise this economic protocol, a summary of the model is provided below to clarify how the economic approach has been developed to further extend the modelling and allow it is to be purposed for economic analysis.

**FIGURE 1:**
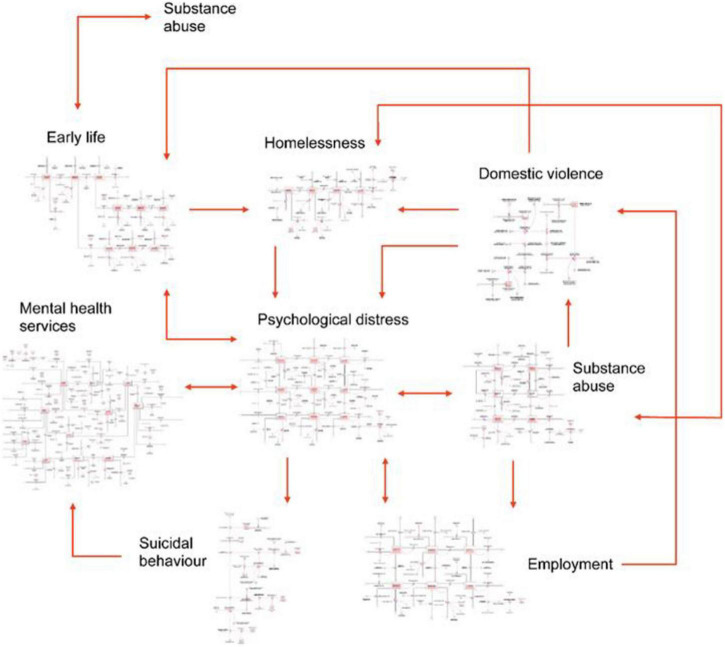
High-level overview of the intended system dynamics model [reproduced with permission (20)].

From a suite of modelling options, the choice of a SD model is considered the most appropriate to combine sufficient scope and necessary dynamics at a population level (25, 39). In generic terms, a system dynamics model is based upon differential calculus and solved by numerical integration. The model’s structural form is visually illustrated in “stocks” (accumulations) and flows (rates of change) and feedbacks between stocks which are driven by model parameters. This becomes a visual illustration of the underlying mathematical model, its complex behaviours (from dynamic interactions) and estimation of both temporal and summative outcomes (Figure 1).

#### Outline of Main Components of the Model

An open population will simulate the changing size and age structure of the local population, accounting for births, deaths, aging, and migration and stratified into age cohorts (0–14 years, 15–24 years, 25–44 years, 45–64 years, 65+ years). Further stratification (e.g., Indigenous status) will be considered conditional upon stakeholder preferences and feasibility regarding data sources. The core of the models is the prevalence of psychological distress in the population and the resultant flows capturing changes in distress levels, self-harm and suicidal behaviour. Distress is measured by the Kessler 10 (K-10) score with the population stratified into three levels (low, medium, high to very high) (42). The main service stocks to be modelled will be determined in the participatory workshops and are expected to include services across patient pathways such as general practitioners (GPs), psychiatrists and allied services, community mental healthcare services, and acute services such as general and specialist hospitals. There is expected to be a wide set of social and economic determinants included, such as employment status, education, homelessness, substance misuse, domestic violence and early life determinants.

#### Main Outcomes

The key clinical outcome measures are likely to include prevalence of psychological distress, mental health-related emergency department presentations and hospitalisations, self-harm hospitalisation rate, and suicide deaths. The key economic outcomes include health utilities (preference-weighted quality of life), years of life lost, quality-adjusted life expectancy, costs (activity-based), budget impacts, productivity loss, and carer time.

#### Parameter Estimation, Calibration, Validation, and Model Function

A detailed list of example datasets that may be used in the study is provided elsewhere, along with details regarding model calibration and validation (39). Model parameterization will draw upon research evidence (including systematic reviews, randomized controlled trials, and cohort studies), survey and service data, and expert consensus. Parameter values for which prior estimates are unavailable will be inferred via model fitting, or calibration. Model validation will compare model outputs with historical trends in key outcome, check face validity of the model and system behaviour among the stakeholder group, and calibrated where necessary to replicate trends. The model will run on continuous time and a time horizon will be chosen appropriate to the needs of stakeholders.

**FIGURE 2:**
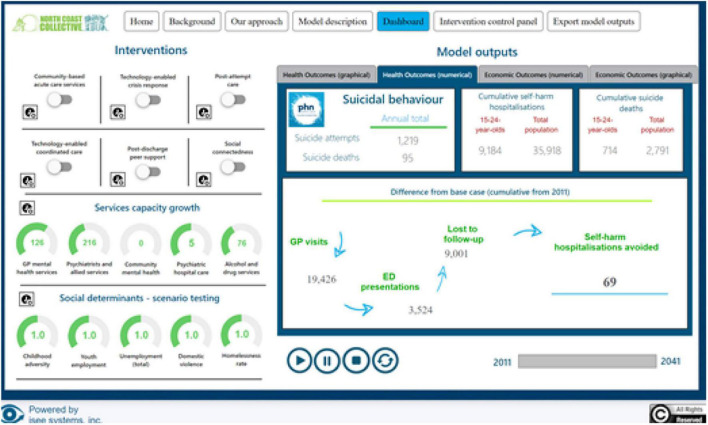
An example model dashboard.

Once developed, the model’s function is to generate projections of system behaviour resulting from the interrelationships and feedback loops represented in the model’s structure. This may, for example, demonstrate the impact of unemployment or homelessness on distress and the use of health services, and the potential negative consequences from capacity shortages in primary, acute care and community settings. In turn, this identifies potential leverage or interventions points and opportunities for close policy coordination, for example. Figure 2 provides an example model dashboard built using Stellar Architect (ISSE systems).^3^

### Purpose and Perspectives of the Economic Analysis

#### Purpose of the Economics and Participatory Workshops

The main objective of integrating an economics component is to equip the SD models to undertake economic analyses and guide investment decisions to improve allocative efficiency. The economist will implement the protocol (e.g., data collection and analysis) continuously through the project with full participation at the three workshops, as follows). Workshop 1 will define the model purpose, scope and sectors included. In turn this determines the economic approach and perspective(s) taken. At Workshop 2, the economist will present the intended approach for feedback and refinement. At Workshop 3, the economist will present the economic work, methods used, how this was integrated into the model, and demonstrate how to generate economic metrics (e.g., return on investment and budget impact). Please refer to the protocol regarding the participatory approach for details.

#### An Integrated Societal Perspective

The approach taken is termed (by the authors), an “integrated societal perspective” (43). The intention is to equip the SD models to conduct and tailor economic analysis to influence decision making across sectors and encourage further multisector coordination. There exists different methodological guidance to conduct economic analysis conditional on, for example, which sector is funding the intervention, the size of the investment and intended value proposition (to minimise costs and/or maximise returns). The intention is to enable the SD model to undertake all main forms of economic analysis to enable the economist to choose from a suite of options, if needed.

For interventions funded by the health sector, economic guidance from health economists (44) recommends taking a “health sector perspective.” Further, the model will be able to take a “societal perspective” to capture both health and non-health impacts, including intersectoral impacts (e.g., social sectors, if modelled), the monetized impact on carers, and productivity loss. This is consistent with economic guidance from Treasury (44, 45) and now required for health sector investments above AUD $10 million within Australia. Within each approach, a health perspective or a societal perspective, there can be different metrics used and each will be generated by the SD model. First, there is a description of the approach to integrating the economics costs and outcomes are integrated in the model (Supplementary File 1 details example data sources).

### Integrating the Economics Into the System Dynamics Model

#### Inputs–Layering-In Costs and Consequences

##### Activity Based Costing Approach

The model will produce estimates of services provision rates over time (numbers of GP consultations, psychiatrist and allied health services, mental health-related ED presentations, psychiatric hospitalisations, etc., per year), which will be used to calculate cumulative activity costs, for example in assessment, treatment, and ongoing management for individuals with psychological distress and suicidal behaviours. The costs considered include: staff time, assets used, materials, and consumables (46, 47).

##### Transaction Costs and Cumulative Costs

There is an important distinction between transaction costs and cumulative costs (46, 47). The former is the result of incurring an acute, time-limited event, and is also termed discrete or one-off costs. This contrasts with the latter, cumulative costs, which refer to the ongoing management of people, for example, who live with psychological distress.

##### Top-Down Approach and Average Unit Costs

To reiterate, the SD models are population level models and reporting will be at the population on aggregate rather than subgroups. Given this structure, the economic approach estimates the average unit costs for a given population and modelled sub-groups where relevant (46, 47). For most person-centred services (e.g., psychologists), this retains validity even at the individual level (e.g., standard fee for service). Discussed below are specific interventions which take a micro-costing approach. All costs will be estimated in constant prices at the latest year available (which will inform the base year used in the model), applying the necessary price deflator, when necessary.

##### Iterative Refinements

If the model is required to report outcomes by sub-groups then the costing approach will become more refined. For example, if age-specific outcomes are important then the costing approach would account for the expected age skew in costs by developing either age specific mean costs or a population distribution. Further, expansions in certain services may encounter capacity constraints and threshold points, where additional and periodic fixed cost investment may be necessary to increase capacity (e.g., new capital investment). As such, costs move from average to marginal, incorporating economies of scale.

##### Health Utilities and Quality-Adjusted Life Years

The SD model simulates and tracks changes in psychological distress using Kessler-10 (K-10) scores. These will be converted to EQ-5D5L scores using a validated mapping procedure to generate “utility decrements” (48, 49). Then, population survival rates within distress states are weighted and summed via integration to generate quality-adjusted life years.

##### Productivity

Living with psychological distress and suicidal behaviour can result in a loss of productivity for those of working age. The main sources of productivity loss will be: time in hospitalisations for mental health related admissions and self-harm, living with moderate and high levels of psychological distress, and death from suicide. These result in a combination of absenteeism (days off work) and presenteeism (at work but less productive than normal). The associated cost of lost productivity will be estimated by applying the average wage, per employee, using regional data (where possible), annualised and adjusted pro-rata to reflect working days lost. For youth, there will be an estimate of days lost from school and the associated time off work for carers. Further, if the SD model estimates the impacts of lost time from school on future employment, these longer-term productivity impacts will be valued also.

A Frictional Cost Approach (FCA) will be taken as default to estimate productivity losses associated with suicide deaths which includes the first 3 months of the estimated productivity loss (50). This is considered to be an approximation to real-world impacts, where, if a person has either died or is continuously absent from work for over 3 months (on average), the employers respond by replacing the worker if possible.

##### Carers

An estimate of the proportion of the population living with psychological distress that require carers will be made, including the hours of care needed. The financial cost will be estimated by the proportion of carers receiving Government support (e.g., a carer allowance) and productivity loss associated with lost employment, also truncated at 3 months. Further, an estimate of total and unpaid carer time will be generated to contrast the potential gaps between the value or opportunity cost of carer time (approximated by the average wage rate) from the actual costs incurred from the proportion of carers claiming eligible allowances. While transfer payments are not normally included in economic analysis the SD models will have this ability to highlight both the financial cost and proportion of carers unpaid incurring opportunity costs. Further, the utility impacts of being a primary carer will also be incorporated by estimating the proportion of the population suffering from high psychological distress that require a primary carer (51, 52), and then applying the appropriate utility decrement (derived from iv, above).

##### Secondary Data Collection

The economist will meet with key local stakeholders (including Primary Health Networks, and Local Health Districts) in advance of the modelling process to initiate procedures and permissions to access relevant service expenditures. Where local estimates do not exist, the default will be to use state level unit costs and apply them to local service activity rates (from the model). There are a range of data sources that will be used with specific values appropriate to each of the eight models. Sources are likely to include, for instance: Australian Institute of Health and Welfare, Australian Bureau of Statistics, Medicare Benefits Schedule, Pharmaceutical Benefits Scheme, Independent Hospital Pricing Authority, peer reviewed journals, and industry reports. The collection, cleaning and analysis of data will continue through the modelling process. The Supplementary File 1 described a range of example datasets and information sources. The selection will be conditional upon the locality of each SD model and the scope of the model as defined in the participatory process (41). No primary data collection will be undertaken.

#### Modelling and Costing the Interventions

##### Selecting Interventions

The participatory process will generate suggestions for candidate interventions to be modelled. The list of interventions to simulate within the model may include, for example, both novel interventions and the scaling-up of existing ones. For illustration, these may include: (a) GP training, to recognise and treat mental illness, (b) post-discharge assertive aftercare, to support people hospitalised for intentional self-harm, and (c) technology enabled care to support better coordination of patient centred care between providers.

##### Modelling the Interventions

**FIGURE 3:**
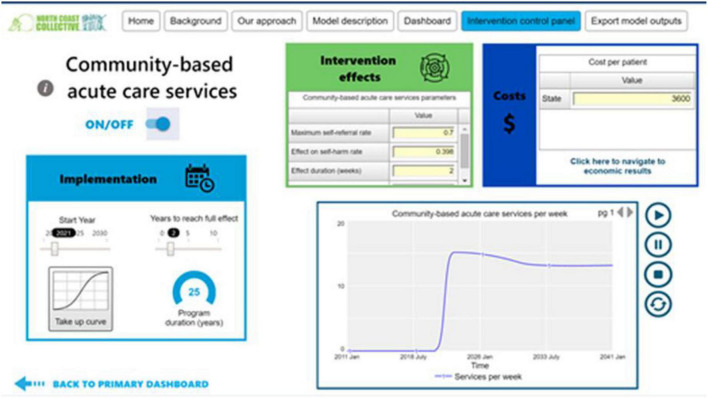
Modelling interventions—an illustration.

Interventions will be explicitly modelled and endogenously run into the core model structure. There is a generic process where three key inputs are required: (a) time—to initiate and stop the intervention; (b) reach and time to scale to the intended size of the eligible population, (c) impact—the effect size of the intervention. These parameters generate S-shaped implementation curves that can be sharpened or flattened conditional upon the values of these variables. Further, the nature of the intervention can become more sophisticated if required to introduce, for example, capacity constraints and delays. Figure 3 provides an illustration of the key inputs to model an intervention that allows modification of the default intervention parameters.

##### Five Types of Interventions Can Be Distinguished

(a) Existing interventions—Prior to the workshops and before the modelling building process, the stakeholder group will be asked, if there are candidate interventions where effectiveness or efficiency could be improved, (b) New interventions with research evidence—to test out the potential impacts in the local system, (c) Adapting existing interventions—to first adjust the model of care to local conditions before simulation, (d) Coupled interventions—such as screening and referral services, (e) Hypothetical or “what-if” scenarios—where the model identifies potential leverage points but where there is not a defined program yet. The model is employed to assess potential benefits of developing an effective service which can be useful to inform the development of new interventions and pilot trials, for instance.

##### Micro-Costing Approach

The interventions (except v) will be defined in detail to permit micro-costings (53). This will distinguish between: (a) “fixed costs”–e.g., capital, set-up costs, (b) “variable costs”—e.g., staff time, materials, and (c) shared costs—if two or more services share resources then costs are not independent. The economist will ensure that effects and costs can adjust accordingly. This approach is intended to permit, where relevant, modelling “economies of scale” (average cost falls), economies of scope (efficiencies from shared resources), and complementary economies, if the impact of one intervention is also conditional on the presence of others. The intended novelties are that in a dynamic system where populations interact with multiple services, we are unlikely to have smooth marginal costs or benefit curves as typically assumed in economic analysis.

#### Generating Economic Outcomes and Valuation Metrics

To reiterate, the economic analysis will take an “integrated societal perspective” tailoring analysis to particular end-users, as appropriate. The model will be equipped to take either a health sector perspective or a societal perspective, and generate relevant metrics, as described next:

##### Cost of Illness—Economic Burden of Psychological Distress and Suicide

First, the model generates projections of business as usual (BAU) including the incidence and prevalence of psychological distress, associated service use, and the resultant adverse outcomes such as hospitalisations for self-harm and deaths from suicide. The economic burden resulting is the summation of health service costs, other sectors (if modelled), the loss of QALYs, loss of productivity, and impacts on carers (QALYs and productivity) (34, 51–54). All impacts will be monetized, including QALYs a described below.

The model projection of BAU generates the simulated counterfactual, against which the impact of investing in interventions and/or rebalancing (disinvestment and reinvestment) can be compared to reduce the cost of illness in BAU. The next stage will be to estimate the impact of the interventions on reducing BUA. As described below, this will involve generating economic metrics of value tailored to the perspective(s) of the analysis.

##### Health Sector Perspective—Cost Effectiveness and Cost Utility Analysis

In this analysis, only costs and outcomes falling on patients and the health sector are considered (44, 55). A cost effectiveness analysis (CEA) assesses the change in net costs to achieve a unitary increase in effectiveness, such as the number needed to screen to avoid self-harm. The information can be used to select more efficient models of care.

A cost utility analysis (CUA) assesses the change in costs to achieve a unitary increase in QALYs. The relevant metric of value for both approaches (CEA and CUA) is the “incremental cost effectiveness ratio” (ICER). This is a statistic that estimates the additional cost to achieve a unitary change in QALYs. For the latter, if the ICER is below AUD $50,000 then the intervention is generally considered to be cost effective (46). A health sector Net Benefit estimate will also be generated by multiplying QALYs by AUD $50,000 where a positive value of considered to be value for money (56).

##### Societal Perspective—Cost Benefit Analysis

A societal perspective will then attempt to value wider impacts aligned with Treasury Guidance (44, 45), including the cost to non-health sectors (e.g., social sectors, if modelled), the monetized impact on carers and productivity loss. The linear sum of a health sector net benefit with these wider monetary benefits then generates an estimate of overall societal net benefit. Treasury departments require this approach in Australia for investments in excess of $10 million. A benefit-cost ratio (BCR) will then estimate the ratio of benefits to the cost of investment (44, 45). The model will also be equipped to generate additional analysis to address particular local decision maker needs, if and when required. This will include cost minimisation and return-on-investment analysis by focussing on costs and potential cost savings.

##### Budget Impact and Distribution of Costs and Benefits

The economic analysis will also generate the overall budget impact from investments, including the size of the investment and flow-on impacts from service use. Further, the model will generate a “who pays” and “who benefits” analysis regarding the distribution of budget impacts on household out-of-pocket expenses, States, Commonwealth, and the wider economy (productivity). This is intended to highlight the potential benefits, where appropriate, of closer policy coordination between sectors to improve mental health and the flow-on benefits to wider societal and economic impacts. For instance, the likely impact of Commonwealth/PHN investments in primary health to prevent acute events and the costs to State funded hospital care.

#### Time Frame and Discounting

The SD model will run on continuous time. As such, the economic analyses can be conducted across any time period considered appropriate. The primary purpose of the model is to inform priority setting (described further below) to impact on population health. This process tends to occur over budgets cycles (e.g., 1 and 3 years) and where planning horizons are typically 10 or 20 years (45, 56). The time frame of the SD models will be decided through the participatory process tailored to decision maker needs. To be clear, the SD models are different from traditional HTA modelling approaches that normally project lifetime impacts on a patient trial cohort. In contrast, the SD models will simulate the open population (births, ageing, deaths, and migration) and directly estimate the population impacts from introducing interventions into the systems, for example. The economic analysis will contrasting impacts from investment between time periods can help inform decision-makers regarding appropriate planning horizons.

In Australia, the choice of discount rate can vary conditional on the preference of the funding agencies. The default rate will be 5% for both costs and benefits when considering a health sector perspective (54) and changed to 7% in other perspectives to follow Treasury guidelines (45).

### Using the Model to Support Economic Decision Making

#### Priority Setting and Investment Portfolios–Identifying What to Do

The SD Model will simulate the local system (population, services, and outcomes) as defined by the participatory group. With a costing and valuation approach described above, this provides the opportunity to model the impacts of different combinations of interventions and take a “portfolio approach” to decision making and investment.

Constrained optimisation will automate the selection of the best combination of interventions relative to an objective function: economic metric to be optimised, intervention options, time frame and budget (35, 37). There will be an ability to change the objective function, such as switching between a health sector perspective and a societal perspective. This is important to tailor economic analysis to the funding audience where the health sector and Treasury (can) have different requirements from economic analysis, as outlined previously.

This approach may be used in tandem with existing priority setting processes, such as Program Budgeting Marginal Analysis (PBMA) (12–15). At the end of the model building process (workshop 3) (41), the economist will engage with stakeholders to gauge interest in using the model in this manner, which may contribute to systematizing the use of the model and economic priority setting in ongoing governance processes. Conditional upon local interest, this may provide opportunities to implement more advanced optimisation routines such as applying a multi-dimensional metric, as used in Multi-criteria Decision Analysis (MCDA) (14, 15). This would permit economic measures of outcomes to be combined and weighted alongside other objectives such as equity.

#### Economic Evaluation–Identifying How to Do It

It is important that the specific intervention(s) chosen, within the priority setting process, are as effective and efficient as possible. A literature review of relevant economic evaluation studies will attempt to identify the most cost effective models of care. This is to reduce waste and release resources to reinvest to further improve population mental health. Where relevant studies exist, the “within-trial” costs and effects of the chosen model of care (e.g., observed over the study duration) can then be run into the SD model to simulate the impacts under local implementation conditions. As new interventions and evaluation studies are conducted, the best performing intervention can be included in an updated optimisation process to rebalance investment portfolios, if needed. Where there is lack of existing evidence regarding the costs to implement an intervention, a “what-if” analysis will be undertaken to simulate the potential impacts. This can then inform the value of conducting pilot trials. Overall, the SD models will be used in a complementary process with traditional economic evaluation, with the latter an input into the former.

#### Uncertainty–Opportunities for Investment in Applied Research

##### Nature of Uncertainty

The model is not intended to be interpreted as prediction tool. The same justification for building a SD model, which is the reality of complex systems, should be applied when using the model modestly. That is, systems evolve, exogenous shocks occur, a model scope can never capture all drivers, and there is never perfect information to inform the model structure, data inputs, modelled relationships or economic information and valuation techniques. The term projection is used here rather than prediction or forecast, which can have connotations that the future can be known with certainty. Rather, the SD model and economic analysis can be used to simulate potential futures and adjust present day decisions to improve the probabilities of better population outcomes. This caveat also applies to the term optimisation where this is a dynamic, not static, approach and conditional upon information available.

##### Forms of Uncertainty Considered

In principle, there are four main types of uncertainty: parameter, heterogeneity, stochastic and structural (57). Ideally, all would be assessed in the current modelling. However, there are practical constraints regarding computational power given the broad scope of the SD models, data, time and resources. The focus will be on parameter uncertainty when simulating the impacts of interventions, as described below. This is considered appropriate given the primary purpose of the model is priority setting at the population level regarding the allocation of resources between interventions. Further, the SD models are intended to be updated over time as new evidence and data become available. Within a Probability Sensitivity Analysis (PSA), parameters will be assigned plausible joint distributions and varied using Latin Hypercube sampling to generate uncertainty intervals (34).

The model is intentionally an aggregate population level model but will be responsive to stakeholder preferences regarding limited model stratification (e.g., by Indigenous status). This will have implications for the economic analysis to refine costs and outcomes, where necessary.

The potential impacts of structural uncertainty will be minimised in two ways. First, the participatory process involves a continuous process of model development, stakeholder feedback, revisions and verification checks to ensure face validity of the final model structure and performance among a diverse range of stakeholders (41). Second, models will be reviewed as new data becomes available providing opportunities for ongoing refinement of model structure.

To inform research priorities, the results from the PSA will be used to rank parameters with respect to the impact of uncertainty of the magnitude of change in the net benefit calculations. This will be done for both health and societal perspectives, noting any inconsistencies in rankings. Overall, engaging with uncertainty is intended to help build the business cases for ongoing investment in data and modelling for public health more generally.

#### Sensitivity Analysis and Stress-Testing: Strengthen the System From Exogenous Shocks

##### Sensitivity Analysis

Sensitivity analysis is different from analysis of uncertainty, from an economic lens. The former is a purposeful choice regarding values and/or what to include in a model, and the latter reflects uncertainty regarding statistical or modelled relationships. The sensitivity analysis will focus on the economic values regarding discount rates, the monetary value of health, productivity and carers. These are set at default levels in accordance with guidance from Health, Treasury and the literature. However, the authors contend that these decisions involve various value judgements containing different normative judgements. It is important that the SD models have the ability to change default assumptions.

The discount rate will have the ability to be varied from between 0 (no discounting) and 10%, in line with economic guidance (45). The monetary value of health can be varied in two ways. The first is to retain a health sector perspective and range the value from AUS $42,000 to $67,000 per QALY (58). The second is to adopt the approach of the Value of a Statistical Life which is approximately $200,000 per statistical life year (59). Productivity will also be changed to a Human Capital Approach to value lost earnings across an expected lifetime, rather than truncating at 3 months assuming replacement in the labour market (50). Carer time can be valued regarding the opportunity cost of time spent using average earnings (for all ages), as opposed to the financial payments currently received and productivity lost from paid employment.

##### Stress Testing and Scenario Analysis

Economic analysis in health and social services typically focusses on marginal allocation of resources between specific interventions to improve efficiency. However, it may be important to also consider if the system is prepared to absorb potential shocks. Exogenous shocks can and will happen, such as recent times of bushfires, floods, COVID-19, and economic recession. There is value in stress testing the systems’ capacity to respond in such scenarios and the implications if there is a lack of preparedness. Stress testing will involve simulating a shock and how this increases the demand for services, the need to adjust service capacities and the opportunity costs of not having slack in the system. This form of prospective modelling also enables decision-makers to run scenarios and test out policy and service response options in a simulated environment before implementation. This can inform optimal responses and steer the system accordingly by investing, where necessary, in appropriate prevention, mitigation and management strategies.

Overall, a key purpose of the economic analysis is also to explore whether there are intervention portfolios to invest in that are robust to the economic perspective taken (e.g., health or societal), statistical uncertainty analysis (e.g., parameter values) and sensitivity analysis (e.g., different normative judgements and discount rates). This may then support the case for sustained funding irrespective of whether decision-makers view the purpose of investment to reduce future acute costs or as investments in improving population health regardless of cost savings.

### Presentation of Model Outputs and User Interface

**FIGURE 4:**
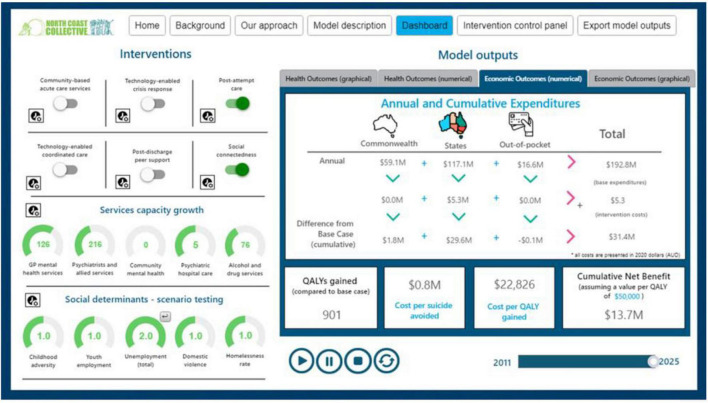
Example dashboard—economic outcomes.

Within the user-friendly Dashboard, there will be specific sections relating to the economic analysis, including an ability to switch on interventions (and adjust timing, roll out), a representation of key economic outcomes including costing (disaggregated by budget holder), valuation measures such as net QALY (health benefits), cost per QALY, and overall net monetary benefits from a health sector perspective, including total QALYs and monetized QALYs minus net costs (Figure 4). Then, using a wider societal perspective, the impacts on productivity and carers will be added. Further, return on investment (ROI) metrics will be generated for both the health sector perspective (monetized health net benefit/investment cost) and the societal perspective (monetized societal benefit/investment cost).

The intention is to empower decision-makers to use the model to achieve insights and inform decision making without having to engage with the model engine itself. As part of the program of work, user guides will be developed to explain how to use the SD models, the meaning of different metrics and generating business cases for funding. The intention is for the research team to maintain close relationships with all study sites to guide model use and for model updating in future research.

## Discussion

This protocol has described the intended approach to economic analysis that will be undertaken as part of a multidisciplinary approach to building and implementing eight SD policy models. The primary purpose is to support priority setting and resource allocation to improve youth mental health outcomes and onward health trajectories (e.g., QALYs) and economic impacts such as productivity. Using a participatory process, the technical model will be developed to simulate the local complex system and the economic components will equip the model to undertake a range of economic analyses. The intention is to support decision-makers to invest in and actively manage the service system to deliver the greatest impacts, and achieve better coordination of investments within an often-fragmented system with multiple funders, providers and end-users. The overall aim of the economics is to help improve allocative and technical efficiency, incorporating equity considerations where necessary.

### Comparison With Existing Literature and Potential Added Value

The existing economic evidence regarding mental health interventions is important and takes a more traditional form of analysis. For example, Australia’s Mental Health Commission after drawing upon the latest academic literature, listed eight interventions in order of modelled cost effectiveness (59). There may be challenges regarding how to interpret and use such evidence to set priorities. For example, the studies used different time horizons, there was no implementation context reported, and valuation was restricted to return on investment. Decision-makers who are tasked with managing budgets over time are left to second guess key operational considerations, such as whether cohort study evidence directly translates into population level impact given local implementation context, what the impacts of investing in combinations of interventions are likely to be, and how costs and outcomes are expected to be distributed over time, and wider metrics beyond cost savings.

The applications of DSM remain limited in health systems and public health (28–34). Further, in infectious disease modelling, where DSM are now used routinely, it has been shown that the cost effectiveness of vaccination programs can vary (60). These studies demonstrate the importance of dynamic modelling to inform strategic investment across interconnected systems to improve population outcomes. An outstanding gap with DSM modelling, overall, is incorporating system-level resource constraints to then identify the optimal combination of investments subject to a budget constraint(s).

### Potential Added Value of a Dynamic Approach to Priority Setting and Integrated Economic Analysis

The SD models coupled with a dynamic approach to economic analysis and portfolio decision making is intended to create an overall macro model of the system, resource constraints (that can be adjusted) and identification of the best investments. This is intended to serve as a modelling framework to support strategic priority setting regarding the best combination of interventions for investment. There are also opportunities to further align different forms of economics analysis within a single model. Regarding economic evaluation, the best performing intervention(s) can be selected and the “within trial” results (rather than final outcomes generated from traditional modelling techniques) can be run into the SD model to project population impacts within the local complex system. Further, the combination of an open population with the economic analysis of “who pays, who benefits” automatically generates a budget impact analysis at both system level and by key sectors (e.g., health and economy) and key stakeholders (PHN, LHD, and Treasury). There is also the opportunity for researchers and decision makers to continuously interact with the model (using the dashboard) to conduct adaptive decision analysis to update the investment decisions over time, if needed.

### Limitations and Further Research

The SD model and economic approach will be subject to practical limitations, including data availability, time to develop the model, and current computation power. First, SD models will take a wide scope to incorporate multiple sectors and sub-sectors. Such breadth entails a trade-off regarding the depth of the model, such as the number of stratifications of the general population and detail regarding service sectors, although interventions will be modelled in detail. Overall, this is appropriate for the selection of an SD model where the primary purpose is strategic policy making across a system (25). It will also present future opportunities to align the SD models with other forms of modelling, such as agent-based models and discrete event simulation which model detailed processes but not the wider system within which an intervention is implemented.

Second, the uncertainty analysis will be mainly focussed on key model parameters following the implementation of interventions as opposed to stochastic or detailed heterogeneity. This is a purposeful decision and considered appropriate for the broad model scope and current computational power available. For context, there are multiple examples where SD models have undertaken in-depth uncertainty analysis where the scope of the model permits (61–63). While HTA models normally have uncertainty throughout all aspects of the model (although rarely structural uncertainty or model updating) a key driver of that uncertainty is the implementation context which is embedded in risk-to-event equations and calibration given the relatively simple model structures. The SD model will explicitly capture such implementation and contextual factors. This is intended to be a more practical approach when the decision challenge is strategic priority setting to manage a system as a whole. Further, results from economic evaluation studies that have conducted in-depth uncertainty analysis to identify cost effective interventions can be read into the SD models to project local impacts. Formal assessment of structural uncertainty is rarely undertaken in health economic models (64) or more complex system dynamics models (the latter due to computational and practical constraints). This is an important area for future development outside of the scope of the current project, however, ongoing efforts will be made to minimise structural uncertainty over time in the models developed.

Third, health utilities and associated decrements may be an underestimate of the impact of mental illness. To reiterate, in this study a pragmatic approach will be taken where the SD model will focus on the dynamics of psychological distress in the general population estimated using K10 scores. Consequently, the economic approach maps K10 scores to utilities which will be undertaken using a validated algorithm calibrated to the routinely used EQ5D5L (49). However, there are novel health economic tools emerging in the United Kingdom for mental health that are likely to be more sensitive to change (65, 66). These may be considered in the future should further testing be undertaken to understand the relative improvements over the EQ5D5L (66). To use these tools in this modelling study there is a need for a mapping algorithm to be developed to convert K10 scores to utilities and using Australian preference weights to ensure validity for the local population. Fourth, the range of data sources will be locally sourced congruent with the eight model locations. However, it is expected that where local data is missing then a higher level of aggregation may be needed and/or calibration required. If such cases arise, these will be reported transparently and would identify opportunities for further data collection.

Going forward, there is expected to be a rich methodological and empirical research agenda to continuously improve models and economics analysis over time, especially with future advances in real-time, big-data coupled with improving computational and analytical capacity. The use of dynamic modelling in public health is comparatively recent compared to other major fields such as meteorology, engineering, defence and finance which benefit from a rich supporting data infrastructure. This permits, for example, the implementation of known techniques, such as Bayesian decision analysis and value of information (67) to analyse the joint uncertainty of multiple interventions simultaneously (67–70). As computational power improves there will be opportunities to apply these techniques for models with broad scope, such as in this program of research.

### Model Updating

The intention is for eight bespoke models to be built across Australia over a period of 4 years. There is a need to ensure that models are routinely updated going forward. Complex systems evolve and so should the models that seek to simulate them. The intention is to initiate a process to help embed system modelling and economic priority setting into governance processes going forward. The methods in this economics protocol, and associated protocols on the participatory approach and the technical blueprint for model development reported elsewhere, are intended to provide an opportunity to be considered, adapted and improved in an international context.

## Conclusion

This protocol describes the generic process of equipping eight SD models to undertake economic analysis, intended to support local priority setting and help optimise implementation regarding the best value mix of investments, timing and scale. Mental health is a complex system, the SD models will capture local dynamics and the economic analysis will purpose the models to demonstrate the value of investing to improve youth mental health. The overall objectives are to improve allocative efficiency, increase mental health and economic productivity.

## Data Availability Statement

The original contributions presented in the study are included in the article/Supplementary Material, further inquiries can be directed to the corresponding author.

## Ethics Statement

This study has been approved by the Human Research Ethics Committee of the Sydney Local Health District (Protocol No X21-0151 & 2021/ETH00553).

## Author Contributions

KL: manuscript concept and drafting. KL, JA-O, LF, AS, YS, GL, SH, and IH: critical revision of manuscript for important intellectual content. All authors contributed to the article and approved the submitted version.

## Conflict of Interest

IH is the Chief Scientific Advisor to, and a 5% equity shareholder in, InnoWell Pty Ltd. The remaining authors declare that the research was conducted in the absence of any commercial or financial relationships that could be construed as a potential conflict of interest.

## Publisher’s Note

All claims expressed in this article are solely those of the authors and do not necessarily represent those of their affiliated organizations, or those of the publisher, the editors and the reviewers. Any product that may be evaluated in this article, or claim that may be made by its manufacturer, is not guaranteed or endorsed by the publisher.

## Footnotes

1
https://www.sydney.edu.au/brain-mind/our-research/youth-mental-health-and-technology/right-care-first-time-where-you-live-program.html

2
https://hnc.org.au/north-coast-collective/

3
https://www.iseesystems.com/
