# Supplementary material for: A Dynamic Approach to Economic Priority Setting to Invest in Youth Mental Health and Guide Local Implementation: Economic Protocol for Eight System Dynamics Policy Models
Source: Front Psychiatry. 2022 Apr 29;13:835201. doi: 10.3389/fpsyt.2022.835201 (PMC9103687; doi:10.3389/fpsyt.2022.835201)
Supplement: Supplementary file 1 [file Data_Sheet_1.pdf]

Supplementary file 1: Generic standard operating procedures (SOPs) to equip eight mental health-related system dynamics models

Purpose of document

This document is intended to accompany the protocol paper, entitled *"A dynamic approach to economic priority setting to invest in mental health and optimise implementation: Economic protocol for eight system dynamics policy models*". The paper describes the rationale and approach that will be taken to equip a series of eight regional system dynamics (SD) models for youth mental health sites to undertake economic analyses to invest in best value interventions. This supplementary document is intended to provide a generic standard operating procedure (SOPs) to support economists who are part of the research team in develop the economic approach for particular models. In effect, the protocol describes in detail the 'what and why' and this SOP describes the 'how, who and when'. In these SOPs there is intentionally a degree of repetition with the protocol paper for exposition purposes so that the SOPs can be a self-contained document.

A series of nine generic stages (and associated steps) will be outlined with illustrative examples. Note, specific costings, outcomes and valuation processes may vary by study sites and particular models. This will be conditional on the participatory process to define the model scope, including the service system to simulation and valuation metrics to use in the economic analysis. First, a summary is provided below regarding the development of the system dynamic (SD) models and the economic components which together combine to operationalise a dynamic approach to economic priority setting and implementation.

Summary of the system dynamics models and economic analysis

A full descriptive of the technical model building process is provided in: *Occhipinti Jo-An, Skinner Adam, Freebairn Louise, Song Yun Ju Christine, Ho Nicholas, Lawson Kenny, Lee Grace Yeeun, Hickie Ian B. Which Social, Economic, and Health Sector Strategies Will Deliver the Greatest Impacts for Youth Mental Health and Suicide Prevention? Protocol for an Advanced, Systems Modelling Approach, Frontiers in Psychiatry, Vol 12, 2021*

The system dynamics (SD) models can be considered in three parts. First, the core structure that simulates the drivers of mental ill-health and pathways of care within the mental health system. This captures the dynamics of: (i) an open population - which changes over time regarding size and composition of the population resulting from births, migration, ageing, and mortality, (ii) an interconnected service system - attempting to meet population needs, including mental health and

social care, and extending to wider drivers of psychological distress such as populations not in education, employment or training (NEET), and (iii) an economic component - to generate costs and outcomes from e.g. psychological distress, self-harm, suicide, life expectancy, quality-adjusted life expectancy (QALYs) and productivity loss. The SD models are calibrated to replicate past behaviour and performance of the system across a range of outcomes and can project forward a base case business-as-usual, i.e. continuing current programs and services as is but accounting for change in population needs over time. Second, specific interventions are modelled in detail and endogenously when permitted by research evidence and/or stakeholder insights. Third, an economic component layers costs associated with modelled mental health services and outcomes into model stocks and flows, as well as the costs associated with implementing additional prioritised interventions and generates a range of economic metrics of value (such as return-on-investment). Integrating costs and outcomes directly into the system dynamics model (rather than using its outputs to undertake a separate economic analysis) permits dynamic analysis of costs and outcomes across time horizons. This then facilitates the development of a dynamic portfolio investment approach to identify the best value interventions, relative to constraints, such as a global budget through optimisation processes. This integrated and dynamic approach to economic analyses can then be employed to generate business cases for new investment and in priority setting exercises to support investment (and disinvestment) decisions to improve population outcomes.

Equipping the model to undertake economic analysis: overview of the nine stages

The economic analysis is embedded within a wider participatory model development process to ensure the principles of co-design apply, as described in: *Louise Freebairn, Jo-An Occhipinti, Kenny Lawson, Adam Skinner, Yun Ju C Song, Grace Yeeun Lee, Samuel Hockey, Sam Huntley & Ian B. Hickie. Participatory methods for systems modelling of youth mental health: An implementation protocol (accepted: JIMR).* Figure 1 is taken from that paper outlines the process of three participatory workshops to develop the model and economic analysis. The entire approach to model building and economic analysis is embedded within the participatory process as described in

There are nine stages that the economic analysis will typically go through regardless of policy context, site location, and the time horizon of the model. The specific details of particular services, costs, outcomes, and the portfolio approach to priority setting will be tailored to each of the eight site models. Each of the nine stages builds upon the preceding one and should be taken in sequential order, although certain steps may overlap for practical reasons such as timing of release of cost data.

The primary purpose of the economics is to equip the SD models to enable strategic priority setting to identify best value combinations of interventions at the population level and a planning horizon that

decision makers wish. There are existing guidelines regarding the conduct of economic evaluation that have different valuation approaches, such as Health Technology Assessment guidelines that focusses on Health Sector (health sector perspective) or Treasury guidelines that widen the approach to also incorporate non-health outcomes (societal perspective). The stages described below here will equip the system dynamics model to undertake analyses that can incorporate all key aspects of these guidelines so that the models can generate economic outputs relevant to different audiences.

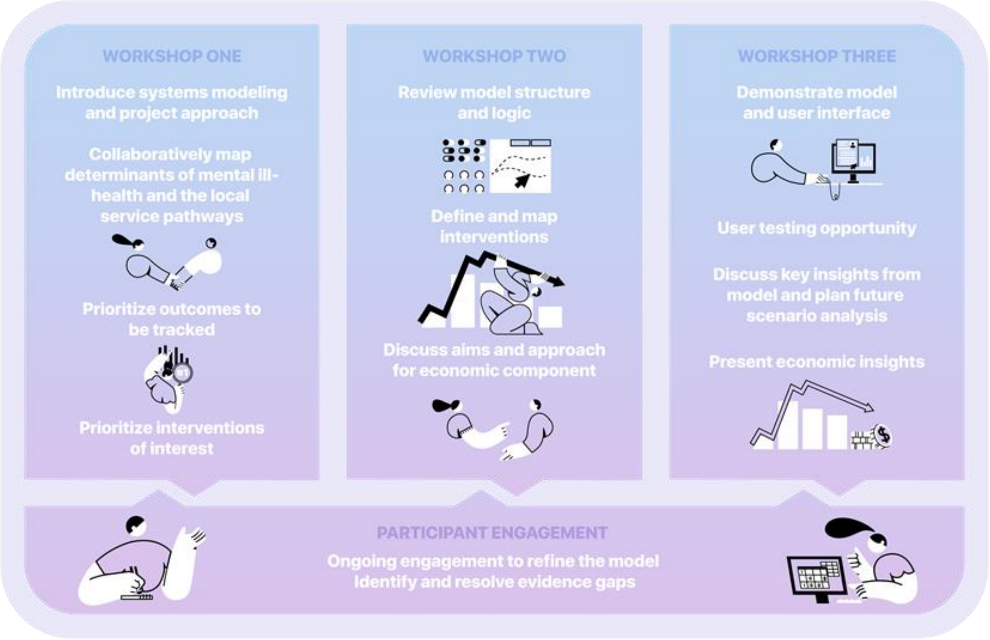

Supplementary figure 1: Participatory process – three workshops

The nine stages are: (1) Pre-model building: Preparing study sites prior to the participatory workshops, (2) Choosing the perspective(s) of the economic analysis and development of an economic protocol, (3) Layering-in costs and economic outcomes (e.g. productivity) into relevant model stocks and flows, (4) Costing the interventions, where possible, (5) Equipping model to generate metrics of value (e.g., return on investment, cost per quality-adjusted life years), (6) Who pays, who benefits' analysis – Identifying opportunities for multi-sector coordination, (7) Developing the economic dashboard, (8) Using the economic outputs to help generate business cases for investment, and (9) Communicating how the model can be used in future economic priority setting exercises to allocate budgets to optimise outcomes.

The rest of this document describes the stages and steps. This is kept at a generic level as the specific details (e.g., programs, interventions) may vary between each of the eight sites. The listed sources of information are illustrative and each study site may have specific sources relevant to particular

populations. Nonetheless, the processes of generating the economic information remain applicable. Each of the eight SD models will have a specific economic protocol developed guided by these SOPs.

Stage 1 Preparing the study sites prior to participatory workshops

There are three visits to each study site prior to the first participatory workshop. The economist will play a role within this process. The purpose is to prepare the primary partner agencies involved in the co-design of the model to understand the role, process, data requirements, and intended value add of the economics component. The economist will attend meetings with the core modelling team and other members of the primary partner agency to provide an illustration of the likely data requirements, form of data needed and timing. The economist should review the site visit agendas to be across details regarding responsibilities, work in relation to wider team and time schedules.

Stage 2: Choosing the model perspective(s) and economic protocol

The economist is embedded within a wider multidisciplinary team involved in model development and the economic analysis is also co-designed within the participatory model building approach. The key guiding principle for the economist is to tailor the economic approach to the purpose and scope of the model. The economics perspective should not be selected in advance of the participatory process. As the scope and purpose of the model becomes clearer, and when the modelling is underway, the economist can then initiate development of the economic protocol to be completed prior to Workshop 2. The protocol should describe the purpose, approach, methods, data, and outcomes resulting from the economic analysis. In short, the economics is intended to purpose-fit each specific models to develop business cases of interventions and undertaken priority setting to allocate limited resource and invest in the best portfolio of interventions.

Steps and timing

As the participatory approach progresses through the Workshops, a reflexive approach is required to retain flexibility, listen to the needs of the stakeholders, direct the conversation when needed to ascertain clarity and revisit the economic approach when necessary.

A stepped approach should firstly understand what perspective(s) the economic analysis needs to adopt. To inform this judgement the economist should first understand the scope and purpose of the model with respect to which decisions is the model intending to support (such as particular policies or program decisions). Secondly, to then identify the Sector(s) responsible for allocating funding, such Treasury, Department of Health, and other Ssctors. Third, to identify which spending Agencies are likely to use the model to inform particular investment decisions (such as Local Health Districts or Authorities). These may be different audiences who operate with particular key performance

indicators and so have specific information needs to inform decisions. Knowing the intended audience with respect to the model outputs will then enable the economist to select an appropriate perspective(s) and purpose the economic inputs, outputs and choice of valuation metrics accordingly.

It is also important to note, that part of the model purpose may be to influence the way decisions and funding allocations may take place, for example, to identify opportunities for closer multi-sector coordination of investments for optimal impact. In these cases, it is important that the economic analysis can not only produce outputs that are aligned with what policymakers are currently responsible for but, when needed, also help create the business case to help align perspectives, budgets, and performance indicators. In these instances, a multi-sector perspective may be warranted, as outlined below.

At Workshop 1, the economics team can help facilitate discussions and present the rationale of the economic analysis and the importance of defining an economic perspective(s). In the field of mental health, self-harm, and suicide research, it is particularly important to use language that is sensitive to the experiences of people who live with mental ill-health, including carers and other support people. From a normative perspective, the economist should be self-aware not to impose a value frame or ethical judgments. Rather, when presenting, it is important to acknowledge lived realities, that the ethical case is of primary significance, and the economics (and modelling in general) is there to support decision-makers to achieve their aims for the benefit of the communities they serve. It is also important to communicate that there are constrained resources (staff available, time, buildings) to achieve outcomes which necessitates priority setting, and part of the purpose of the economic analysis is to help produce robust business cases to advocate for additional resources when warranted.

At Workshop 2, the economics team presents on the intended approach to the economic analysis for feedback. Following Workshop 2, the details of the interventions being modelled becoming clearer and the main body of work for the economics team begins.

It is anticipated that SD models may combine a societal perspective (e.g., include all major costs and benefits, where data permits) and also report a specific health sector perspective. Combining perspectives can be complementary and help make the case for sustainable funding across political cycles if policymakers have different preferences regarding what public services are aiming to achieve (e.g., to save future costs and/or invest in health and wider outcomes such as economic participation).

Following Workshop 2 the economist can make revisions to the economic protocol given stakeholder feedback on the presentation at Workshop 1. The rest of this SOP lays-out the generic steps that the protocol would operationalise and tailored to each of the eight study sites.

Stage 3: Layering-in economic information into the model & estimating business-as-usual

As described earlier, the SD model will have defined components, such as open population, a psychological distress component, mental health services component, wider social services component, among others. Within each component there will be defined model stocks which can represent particular services. For example, for the health sector this may include, but not limited to, stocks such as primary care (general practitioners), allied services (psychologists, psychiatrists), emergency department presentations, and hospital admissions. The model then estimates the populations flowing into each stock per unit of time, time residing in stocks and outflows to other stocks and/or components, or death.

Steps and timing

With respect to costing services in business-as-usual (i.e. not considering new interventions at this stage) an activity-based costing approach will be taken. The default method will be a top-down costing approach using the latest year available to source costs. Costs (and outcomes) will be discounted to the latest year consistent with base year of the SD model. So, for instance, the model may have base year of say 2021 however the latest price deflators are typically 2 years lagging and so prices would be 2019. This is aligned with standard practice in economic costing. Overall, constant prices are important so that when costs change over time this is driven by volume rather than nominal inflation.

The model stocks will be defined at an aggregate level (e.g. general practitioners, employment) rather than specific models of care or sectors of employment. The focus of model reporting is also at the population level rather than individual subgroups, although there may be a limited model stratification (e.g. Indigenous and non-Indigenous). As such, the costing takes a similar aggregative approach in top-down costing. To be clear, at an individual level costs are typically skewed (e.g. elderly typically have greater length of stay in hospital). Note, when considering specific interventions and models of care a detailed micro-costing approach will be taken, described below.

The aim is to develop an average cost, per person, per unit of time, transitioning through the relevant stocks. The economist must understand the definition of each model stock and which service or array of services are included. The economist then sources the relevant expenditures, estimates the costs and provides the data in user-friendly format to the modeller (e.g. CSV file). In situations where there are population costs but not per person costs, and/or when costs related to annual expenditures then these may be converted to a per person per unit of time by the model itself, given the population flows are being simulated in continuous time. Costs are then attached to flows when there are discrete costs (e.g. new GP consultations) and consequently overall population costs accumulative within

stocks. Alternatively, for on-going costs (e.g. productivity losses from unemployment) these are attached to stocks directly and the population residing within a stock is then multiplied by the appropriate cost.

When necessary, care must be taken to distinguish between operational or variable costs (VC) such as staff salaries and materials from capital and managerial (service coordination), which we call fixed costs (FC) for exposition (fixed over a period of time before e.g. depreciation and replacement). Costs that have both components may then be subject to economies of scale (i.e., as the population receiving the service increases the average per person cost falls). When necessary, the economist needs to then define threshold points where new fixed investment is required (e.g. when client population exceeds service capacity). In these cases, there a simple stepped cost function is defined with the modeller.

The calculations are typically simple with an emphasis on sourcing the right data and level of aggregation for the particular model.

Average costs 1 = Total expenditure per unit of time (FC + VC) / total modelled population

Average costs 2 = Weighted sum of specific expenditure per unit of time (FC + VC) / total modelled population

Example 1: For illustration, it is anticipated that there will a model stock referring to 'allied mental health services' and it is important to account for the bundle of specific services that may reside within that stock, such as psychologists, psychiatrists, mental health nurses, occupational therapists etc. These providers operate a fee-for-service model with standard fees publicly available from Medicare Benefits Schedule (MBS) and Pharmaceutical Benefits Schedule (PBS). Further, there is also public data available regarding total expenditures across categories at national and regional levels. Therefore, to develop an average cost per person a simple ratio is calculated of total expenditure divided by total population that received that service. If a stock aggregates across different services, then it will be necessary to generate weighted service costs by population(s) using each services, with data also publicly available.

If the model perspective goes beyond the health sector (as expected), then costing needs to source relevant information accordingly. For example, if the model scope extends to the social drivers of mental health outcomes such as homelessness and domestic violence, this then has cost impacts for Family and Community Services (FACSs), Police and the justice system with a mix of State and. Commonwealth funders. Again, the same basic principles of costing apply.

With respect to uncertainty regarding economic inputs (e.g. costs, utilities) the focus is on parameter uncertainty, as explained in the protocol paper. This may extend to heterogeneity if stakeholder have a preference for stratified models (e.g. Indigenous, non-Indigenous). If so, then costing will be required to be specific to different group. Uncertainty of economic inputs can be accounted for by either by using reporting uncertainty intervals around means estimates from research studies where available, or otherwise a default uniform distribution of +/- 20% can be applied. For the development of the model structural or stochastic uncertainty is not included for practical constraints, mainly computational power and time. The scope of the SD models are anticipated to be much wider that either traditional economic models or most SD models where all forms of uncertainty can be more easily accommodated. This is considered appropriate given the purpose of the model is strategic priority setting at the population level.

Sources of information:

This will likely include, but not limited to:

(1) Expenditures on primary care and specialist allied mental health services – Medicare Benefits Schedule. This will be available from The Australian Institute of Health and Welfare, https://www.aihw.gov.au/reports/mental-health-services/mental-health-services-in-australia/report-contents/expenditure-on-mental-health-related-services/specialised-mental-health-services-expenditure

(2) Expenditures on pharmaceuticals – Pharmaceutical Benefits Schedule. This will be available from The Australian Institute of Health and Welfare, https://www.aihw.gov.au/reports/mental-health-services/mental-health-services-in-australia/report-contents/expenditure-on-mental-health-related-services/specialised-mental-health-services-expenditure

(3) Community mental health services – seek permissions to access specific program data from Primary Health Networks and Local Health District

(4) Emergency Department and hospital admissions – seek permission to access local LHD expenditure data including average length of stay (LOS) for admissions. If unavailable, then proxy default is the Independent Hospital Pricing Authority (IHPA) that has both national average and can be adjusted to particular areas using National Weighted Activity Unit (NWAU) calculators.

https://www.ihpa.gov.au/what-we-do/pricing/national-weighted-activity-unit-nwau-calculators/nwau-calculators-2021-22

(5) Ambulance – each state has its own costing structure, including co-payments and exemptions. The relevant Ambulance authority can be contacted. Where costs are also conditional on km travelled, an average travel duration is required based on local size, hospital catchment areas. The following link provides an example for NSW and associated regions https://www.ambulance.nsw.gov.au/our-services/accounts-and-fees. This should then be weighted by average admission by ambulance as opposed to alternative transport options https://www.aihw.gov.au/reports/mental-health-services/mental-health-services-in-australia/report-contents/hospital-emergency-services/patient-characteristics.

(6) Potential non-health service costs – Conditional upon the scope of the model, it may be necessary to cost the impact of homelessness, domestic violence and criminal justice, for instance. The Productivity Commission undertook a wide review of mental health and expenditures in 2019 which can be direct source and/or a focal point to update the estimates made by following sources in that document (see below). Otherwise, additional literature reviews (academic and grey) may be needed to update these estimates. For instance:

https://www.pc.gov.au/inquiries/completed/mental-health/report/mental-health-appendices.pdf

https://www.ahuri.edu.au/__data/assets/pdf_file/0007/2032/AHURI_Final_Report_No218_The-cost-of-homelessness-and-the-net-benefit-of-homelessness-programs-a-national-study.pdf

https://www.dss.gov.au/sites/default/files/documents/05_2012/cost_of_dv_to_australian_economy_i_1.pdf

https://www.dss.gov.au/sites/default/files/documents/05_2012/cost_of_dv_to_australian_economy_ii_2.pdf

Utilities and Quality-adjusted life years

In addition to costs, the model will estimate quality-adjusted life years (QALYs). With respect to quality-adjustment, the SD models will first estimate the populations background quality of life using population norms. This is a linear statistic that for expositions range from 0 (dead) to 1 (perfect health). In practice, different measures can be truncated e.g., above 0 (e.g. 0.29 for SF-6D) or below zero to indicate a state considered worse than death (EQ-5D). Norms can then be applied by age groups if the model requires (e.g. the dynamics of the open population). Next, the utility decrement is estimated which is the reduction in background quality of life from living in different states of psychological distress. Distress will likely be measured by the Kessler-10 (K10) and converted to the relevant health utility values by applying a conversion algorithm (see below and source 4). This can either be the actual K10 score or if the modeller chooses to group the population into categories (e.g. low, moderate, high-

very high levels of distress) then the economist will discuss with the modeller what mid-point value of the K-10 to use and generate the equivalent health utility value using the mapping algorithm. Health utilities are then used to weight survival in the model and summed across time to generate cumulative QALYs. Again the focus is on parameter uncertainty.

Health utility = population norm – utility decrement

Utility decrement = Exp(3.52220−0.01382*AGE − 0.06476 × K-10) / (1 + exp(3.52220−0.01382 × AGE − 0.06476 × K-10))

Quality adjusted life years = Cumulative survival * (health utility – decrement)

<u>Sources of information:</u>

(1) Population norms: Viney R, Norman R, King MT, Cronin P, Street DJ, Knox S, Ratcliffe J. Time trade-off derived EQ-5D weights for Australia. Value Health. 2011 Sep-Oct;14(6):928-36. doi: 10.1016/j.jval.2011.04.009. Epub 2011 Aug 4. PMID: 21914515.

(2) K10: Kessler, R.C., Andrews, G., Colpe, .et al (2002) Short screening scales to monitor population prevalences and trends in non-specific psychological distress. Psychological Medicine, 32, 959-956.

(3) Using K10 scores: Andrews, G., Slade, T (2001). Interpreting scores on the Kessler Psychological Distress Scale (K10). Australian and New Zealand Journal of Public Health, 25, 494-497

(4) Utility decrements mapping: Gamst-Klaussen T, Lamu AN, Chen G, Olsen JA. Assessment of outcome measures for cost-utility analysis in depression: mapping depression scales onto the EQ-5D-5L. BJPsych Open. 2018 Jul;4(4):160-166. doi: 10.1192/bjo.2018.21. PMID: 29897028; PMCID: PMC6034447.

Productivity

The impacts of distress on productivity may be of interest to the stakeholder group and/or the potential policy audiences (e.g. Treasury). Productivity can be lost as a result of days spent in hospital due to self-harm and suicide behaviour, death from suicide, and as a result of living in the community with moderate and high-very high psychological distress. The productivity impacts can be divided into days lost from absenteeism (not working) and presenteeism (working at less than normal productivity). The value of each day lost, adjusted pro rata, is the average wage applicable for regional focus of the model. For death, the lost productivity is capped at three months using a Frictional Cost Approach with the assumption of replacement from the labour market, on average. Sensitivity analysis can then be undertaken to explore the impact of using the Human Capital Approach which removes the three-month cap. The model will be equipped to generate both FCA and HCA, with the

former as the default. There are four main instances of productivity loss, which may be extended conditional upon model scope.

*Hospitalisation = Employment rate * [X days in hospital * average wage rate (pro rata)]*

*Death = Employment rate * [X days of lost working age years * average wage rate (pro rata)]*

*Moderate distress = Employment rate * [X days * average wage rate (pro rata)]*

*High to Very High distress = Employment rate * [X days * average wage rate (pro rata)]*

Further, if the model is explicitly simulating population flows between employment, education and training, unemployment and underemployment, then there are potential productivity impacts incurred. As such, the economist should provide an average wage rate appropriate to the regions. If underemployment is a model stock then this requires an estimate of % time worked (e.g. 60% of a full-time role) to weight the average wage rate accordingly. Further, psychological distress in youth may result in lost days from school and/or increased work absenteeism from guardians to perform caring duties. If the model estimates the extent of school absenteeism then the productivity loss from guardians will be estimated also. Finally, if the model estimates the impact on prolonged school absenteeism on future employment prospects the economic analysis can then value the associated lost productivity also. For each of these a review of the literature will be necessary to select appropriate estimates and/or generate plausible assumptions to be shared with stakeholder group in model documentation.

Sources of information:

(1) Hospitalisation from self harm - Request data from Local Health District regarding costs, including length of stay. Otherwise, the economist needs to generate costs a proxy of which can be, for example:

https://journals.sagepub.com/doi/pdf/10.1177/0004867417717797?casa_token=QFejI8wmdlgAAAAA:_K_ZtXu8kVnd6H3ZlChvrmHakYzMMcEXgK-B6MVZ_gKIJHIgPZeeO2KvsSpJbOQA1yLs53u8SqOs

(2) Productivity – days of absenteeism and presenteeism due to medium and high to very high psychological distress: https://www.pc.gov.au/inquiries/completed/mental-health/report/mental-health-appendices.pdf

(Soured from: National Health Survey 2017-18 TableBuilder, Cat. no. 4324.0.55.001, Canberra.)

(3) Productivity - average earnings per week: Australian Bureau of Statistics :

https://www.abs.gov.au/statistics/labour/earnings-and-work-hours/average-weekly-earnings-australia/latest-release

https://www.abs.gov.au/statistics/labour/earnings-and-work-hours/personal-income-australia/latest-release#local-government-area-median-total-income

https://www.abs.gov.au/statistics/labour/employment-and-unemployment/labour-force-australia/mar-2021#hours-worked

(4) Frictional Cost Approach and Human Capital Approach:

Pike J, Grosse SD. Friction Cost Estimates of Productivity Costs in Cost-of-Illness Studies in Comparison with Human Capital Estimates: A Review. Appl Health Econ Health Policy. 2018;16(6):765-778. doi:10.1007/s40258-018-0416-4

Carers and informal care

If a societal perspective is adopted, conditional upon model scope, it will be important to account for carer time, financial support and potential productivity lost also. This requires an estimate of the proportion of the population requiring care by category of distress (e.g., moderate, high to very high), and average days of care required (pro rata). This estimate is then converted to a dollar amount by estimating the proportion of carers who receive Government financial support (e.g. carer allowance). Further, the costing analysis will also estimate the opportunity cost of time, irrespective of current levels of financial support, using the average earnings (pro rata). The difference between approaches can serve as a proxy for the value of unpaid carer time. Economic analysis does not normally include 'transfer costs' however the model will be equipped to estimate these. This provides an opportunity to estimate i) the proportion of carers receiving payment from those eligible, and ii) contrast current financial support with the opportunity cost of time. Both may be illustrate of the potential to further support carers.

At the time of writing, there was strong data on the care needs for people in high to very high distress but less so for those in moderate distress. Further, the focus was also on primary carers as the data is more reliable, notwithstanding that those in the very high distress category may need more than one carer. Therefore, to be conservative, the estimates should begin with those with high to very high distress and resurvey the literature when required.

*Carer financial support = High to very highly distressed population * Prob of carer * Carer days * carer allowance*

*Carer opportunity costs = High to very highly distressed population * Prob of carer * Carer days * average earnings*

Sources of information:

(1) Carer hours for high distress: https://www.mindaustralia.org.au/sites/default/files/Mind_value_of_informal_caring_full_report.pdf

(2) Carer supports and valuation: https://www.pc.gov.au/inquiries/completed/mental-health/report

Potential service capacity constraints and impacts on costing

The key focus of the model will be interventions (discussed below), however part of the model function will also be to estimate how the demand and supply of core services change. It is important that the economist discusses with stakeholders the potential implications for costing if the model were to simulate an expansion of services. For example, while the majority of costs will be fee-for-service to which the application of average costs still holds, it may be the case that additional capital costs are incurred. A potential scenario, for example, may be if new infrastructure or buildings are required to house more health service staff or whether existing facilities are sufficient to handle additional throughput. In the event of such costs being incurred this must be accounted for. As such, there would be a step-change in average costs which then falls to a new level as economies of scale spread capital cost over higher patient volumes.

Defining 'who pays'

It is also important that the economist identifies where the cost burden falls and the modelling team then insert identifiers into the stocks to keep track of the distribution of impacts across different payers. Australia operates a complex health system with an interconnected mix of financing. The focus of the model is the public sector. To simplify complexity, four 'payers' are identified in a 'budget holder approach', namely: State, Commonwealth, Households and Economy (productivity). States have the responsibility for hospitals expenditures even though approximately 45% of funding is sourced from the Commonwealth. The Commonwealth also funds primary health care, allied health services and certain community services directly (e.g. headspace services), through Primary Health Networks (PHNs), and through the MBS. Some primary and allied health services can require patients to contribute co-payments which tend to be 75-85% of the MBS / PBS scheduled amount. However, it is vital to account for the latest exemptions, threshold payment ceilings, and the proportion of General Practitioners who bulk-bill hence not requiring co-payments by patients. These can all be used to generate a % weight against the co-payment to adjust this downwards. For example, in generating

average co-pays this would be weighted by proportion bulk billed (if primary care), co-payments taken from MBS/PBS schedules, and capped at the threshold level. Further, the impacts on carers are also included under Households. Finally, the productivity costs are allocated to the economy.

Note, this analysis from the budget holder perspective as the key decision-maker(s) regarding expenditures of the budget. It is recognised that there is a circular flow of income and expenditures between, for example, Commonwealth and States, households and economy, and so forth. The budget holder perspective in the 'who pays' 'who benefits' analysis is a pragmatic approach to influence decisions taken by the budget holder. Note, transfer payments are excluded, in keeping with usual practice in economic analysis (with the exception of carers above).

Sources of information

(1) Commonwealth funding of State hospitals: https://www.aph.gov.au/About_Parliament/Parliamentary_Departments/Parliamentary_Library/pubs/rp/rp1819/Quick_Guides/FundingPH

(2) Out of pocket costs https://www.health.gov.au/health-topics/private-health-insurance/what-private-health-insurance-covers/out-of-pocket-costs

(3) Specific costs https://www.health.gov.au/resources/apps-and-tools/medical-costs-finder

(4) Bulk Billing behaviours https://www.health.gov.au/ministers/the-hon-greg-hunt-mp/media/medicare-bulk-billing-rates-continue-to-grow

(4) PBS thresholds, exemptions, overview https://www.pbs.gov.au/info/healthpro/explanatory-notes/front/fee

Business-as-usual and economic burden of mental illness

Once the model is developed, calibrated to historical data and the economic costs and outcomes are layered in, an economic burden of disease analysis can be generated. For example, the model can project estimates of the total burden of psychological distress on QALYs, service costs, productivity, and on carers or other support people. This serves as both the economic burden of mental illness and also the modelled counterfactual against which the impacts of new investments in program and services (or disinvestment is compared against) can be measured.

Stage 4: Costing the priority list of interventions and 'what-if' analysis

As described in the protocol paper for the participatory process, stakeholders will identify and define a priority list of interventions (e.g. 6-8 candidates). This may consist of scaling-up existing interventions

or introducing new interventions and may also include different forms of interventions including legislation, public health campaigns, training of professional staff, service efficiency innovations (e.g. technology enabled care) and service offerings such as better access to required care in for those in distress and post-self harm.

Further, it will also be important for stakeholders to consider whether there are opportunities to improve the effectiveness and/efficiency of delivery of existing services before turning to opportunities to invest more resources. That is, there may be opportunities to also disinvest in existing services that may lack effectiveness/efficiency to then free-up resources (e.g. staff time) to be reinvested elsewhere to improve overall mental health outcomes. If so, then there are opportunities for the model to endogenize candidate services for disinvestment within the business-as-usual simulation. This prepares the ground for an economic optimisation exercise to improve the current functioning of existing services as well as identifying new best value investments with additional resources.

The role of the economist is to participate in discussions at the three workshops and to emphasise that interventions can only be properly costed if the model of care or service delivery is well-defined. If so, a micro-costing approach will be the default. It is important that close communication with the modelling team is ongoing to ensure consistency between how interventions are defined and costed and how they are integrated into the model.

Regarding the component parts of an intervention, it is important to consider when relevant the associated fixed costs (FC) and variable costs (VC). Fixed costs refer to start-up costs, including, for example, capital, management, and coordination costs that are invariant to the size of the population. Variable costs include, for example, staff fee-for-service, and overhead costs that may be driven by population numbers. These are important to capture as the combination of FC and VC drive economies of scale. Strictly speaking, even fixed costs are time dependent (e.g., capital deprecation) and capturing this identifies time points where further investment is needed (e.g. capital replacement). In such cases, there is not a single average cost but a step-function that repeats as a service or intervention increases in scale.

It is also important to investigate if an intervention has shared costs with other interventions (new or existing). If so, these economies of scope need to be accounted for by weighting and allocating the costs to respective interventions. This generates additional synergies that a portfolio approach to investment can capture.

There is not a single costing formula to apply but rather the economist will apply these principles. Importantly, the interventions will be endogenously modelled and time to implementation, scale, roll-out period, and maturation will all be adjustable. Therefore, the economist needs to provide the modeler with the detailed breakdown of what specific intervention components drive costs and which sector(s) is responsible for funding the interventions.

A key feature of SD modelling and the participatory process is for all stakeholders and the modelling team to learn from the model itself. That is, once the model is constructed the simulations can often present novel insights regarding the way in which populations interact with components of the system, the interdependent behaviour of different parts of the service system, and the non-linear outcomes that can result. When analysing the sources of behaviours, key leverage points in the model may be identified that can elicit significant impact on the simulated outcomes of interest.

In an attempt to influence the direction of these leverage points, often there is only a thematic, rather than well-defined, interventions that can be costed. A 'what-if analysis' can use the model to hypothecate the potential flow-on consequences on outcomes and costs if an intervention could be developed to modify the leverage point in a positive direction. The value of this approach is to direct efforts to develop novel interventions to be using in pilot trials and evaluated before large scale implementation. That is, the 'what-if' analysis can be used to generate business cases to fund applied research to develop, test and implement interventions going forward.

Sources of information:

(1) Costing methodologies Top-down and bottom-up (micro) – overview and guidance:

Chapko, Michael & Liu, Chuan-Fen & Perkins, Mark & Li, Yu-Fang & Fortney, John & Maciejewski, Matthew. (2009). Equivalence of Two Healthcare Costing Methods: Bottom-Up and Top-Down. Health economics. 18. 1188-201. 10.1002/hec.1422.

Olsson, T.M. Comparing top-down and bottom-up costing approaches for economic evaluation within social welfare. Eur J Health Econ 12, 445–453 (2011). https://doi.org/10.1007/s10198-010-0257-z

(2) Microcosting review

Xu X, Lazar CM, Ruger JP. Micro-costing in health and medicine: a critical appraisal. Health Econ Rev. 2021;11(1):1. Published 2021 Jan 6. doi:10.1186/s13561-020-00298-5 https://www.ncbi.nlm.nih.gov/pmc/articles/PMC7789519/

Stage 5: Generating economic metrics: The value proposition for investment

It is expected that the purpose of all eight models will be to influence decision-makers across different sectors and also at Treasury level. That is, the analysis will seek to influence funding allocation decisions to sectors (Treasury) as well as influence decisions within spending sectors (e.g. Health) regarding what interventions to invest in.

Health Sector Perspective

The economic analysis within the health sector has guidelines regarding what metrics should be generated and the methods of generating these. The three most prominent are cost effectiveness, cost-utility, and net benefit. The model should be purposed to generate all three. The value proposition is to identify the interventions that can generate the greatest health impacts relative to a budget constraint.

<u>Cost effectiveness analysis</u>

Effect (non-monetized measure of effect e.g. self-harm rates) /

net cost (cost of intervention(s) + flow on cost impacts in system)

= cost per effect (e.g. suicide avoided)

Informs which intervention or combinations of interventions are most technically efficient (lowest cost per effect). This informs the best models of care and frees up resources to be reallocated elsewhere to improve outcomes further.

<u>Cost utility analysis</u>

QALYs (quality-adjusted life years) / net cost (cost of intervention(s) + flow on cost impacts in system) = Cost per QALY

QALYs provide a generic outcome intended to enable the comparison of investing in mental health to increase QALYs versus other interventions areas such as cancer or cardiovascular disease. This can be used by the health sector to allocating funding between different areas across the health system overall. A cost per QALY of $50,000 or under is considered value for money.

<u>Net benefit</u>

*QALYs * $50k (willingness to pay for 1 QALY) - net cost (cost of intervention(s) + flow on cost impacts in system) = $*

Net benefit can be more easily used in an optimisation process as it is a single statistic to be maximised as opposed to the ratio cost per QALY. In addition, the following financial metrics can be used to assess affordability.

<u>Return on investment</u>

∑ (Cost savings) / ∑ (intervention costs) = ratio

If the ratio is: above 1 = candidate for investment; 0 = neutral, less than 1 = poor investment

<u>Budget impact</u>

∑ (Cost savings – intervention costs) * eligible population = $

This estimates the affordability of the intervention today, irrespective of the ROI. That is, an intervention may have a significant ROI over time, but given limited resources is unaffordable in the short term. Note, this is with reference to real resources (e.g. available of staff) as opposed to financial budgets which can be changed. That is, even large increases in budget may not make any difference if for example there are not sufficient staff available to implement an intervention. Nonetheless, the analysis can reveal potential opportunities for training for example to improve workforce over time. As the SD models simulate the open population the budget impact can be automatically generated.

Societal Perspective

<u>Cost benefit analysis</u>

Net benefit from health sector perspective + net costs from other sectors

+ productivity + carers = $

This can be considered the widest or most inclusive of the metrics where the value proposition extends to non-health impacts. A pragmatic approach is taken to account for the impacts across sectors and where possible productivity losses and carer impacts. No primary analysis is conducted (e.g. discrete choice experiments). This analysis is appropriate for Treasury decision when allocating funding between health and non-sectors, where business cases requiring new funding may be competing with, for instance, business cases from other sectors (e.g. education, housing). This essentially takes a multisector perspective, conditional upon model scope. The authors term this approach an integrated societal perspective where both the overall societal impacts is reported and the distribution of costs and benefits between sectors, households and the economy.

The intention is to report a net benefit value to provide comparability, inclusive of the net benefit from the health sector perspective. In addition, the analysis would estimate the benefit-cost ratio and budget impacts, following a similar approach as above but widen to include non-health impacts.

Time frame, discounting, constant prices, sensitivity analysis

The time horizon of the model can be any period that the participatory group requires. It is important to reiterate that this model and the economics is purposed for priority setting to achieve population outcomes over planning horizons. The model generates aggregate mental health outcomes for a given population catchment with an open population that changes over time due to births, migration, aging and death), rather than lifetime impacts from a patient cohort. The most common long term planning horizons tend to be 10 or 20 years. Nonetheless, the model can generate cumulative outcomes across any time period. Model flexibility allows variation in the discount rate for scenario testing with the default discount rate of 5% (health sector perspective) and ability to increase to 7% (Treasury, societal perspective). This sensitivity analysis should be extended across the economic analysis to be switch from default values concerning valuing QALYs, carers, and productivity. In the core modelling, these are respectively, $50,000 per QALY, those taking Carer allowance, and Frictional Cost approach. Sensitivity analysis will vary each so that QALY value can range from $42,000 to $67,000, carer time irrespective of receiving Carer allowance and Human Capital approach to productivity loss.

Sources of information

(1) Health economic guidelines

Husereau D, Drummond M, Petrou S, Carswell C, Moher D, Greenberg D et al. Consolidated Health Economic Evaluation Reporting Standards (CHEERS) statement BMJ 2013; 346 :f1049 doi:10.1136/bmj.f1049 https://www.bmj.com/content/346/bmj.f1049

(2) Treasury economic guidelines https://www.treasury.nsw.gov.au/finance-resource/guidelines-cost-benefit-analysis

(3) Societal perspectives for mental health - Duevel, J.A., Hasemann, L., Peña-Longobardo, L.M. et al. Considering the societal perspective in economic evaluations: a systematic review in the case of depression. Health Econ Rev 10, 32 (2020). https://healtheconomicsreview.biomedcentral.com/articles/10.1186/s13561-020-00288-7

(3) Health sector deflators https://www.aihw.gov.au/getmedia/a5cfb53c-a22f-407b-8c6f-3820544cb900/aihw-hwe-80.pdf.aspx?inline=true

(4) Other deflators https://www.abs.gov.au/statistics/economy/price-indexes-and-inflation

(5) Value of a QALY https://www.sciencedirect.com/science/article/pii/S0277953618303095

Stage 6: 'Who pays, who benefits' analysis – Identifying opportunities for multi-sector coordination

Once the model is equipped to estimate the impacts of interventions to change the BAU, the model will generate if and how the distribution of impacts change. The impact of interventions then may increase the costs on some payers and reduce the burden on others. For instance, primary prevention (e.g. GP screening and intervention) is funded from the Commonwealth and may then reduce self-harm and acute care which fall on State funded hospitals. To reiterate, the analysis from a budget holder viewpoint.

The rationale for the 'who pays, who benefits' analysis is to help identify potential opportunities for multi-sector coordination to improve overall system outcomes. At present, individual sectors can often operate in silos, only concerned with their own immediate budgets and resulting in cost-shifting. Only taking a health sector perspective may risk reinforcing silo behaviour. This analysis may help inform 'whole of government' decision-making if sectors can be funded to account for such distributional impacts.

Stage 7: Developing the Dashboard

Once the model is functioning and producing the required information, the economist should work alongside the modeller to purpose-fit the dashboard to deliver dynamic economic analyses. Once the formulas are embedded in the programming of the model, all metrics can be automated. The model dashboard and documentation will then summarise what each metric means, its purpose and how users of the model can generate associated business cases for investment.

Within the user-friendly dashboard, there will be specific sections relating to the economic analysis, including an ability to switch on interventions (and adjust timing, roll out), a representation of key economic outcomes including costing (split by payer), valuation measures such as cost per QALY and overall net benefits including productivity and carers (see figure 2 which represent the health sector perspective). The intention is help empower policymakers to use the model without necessarily being across the technical details of the model engine itself.

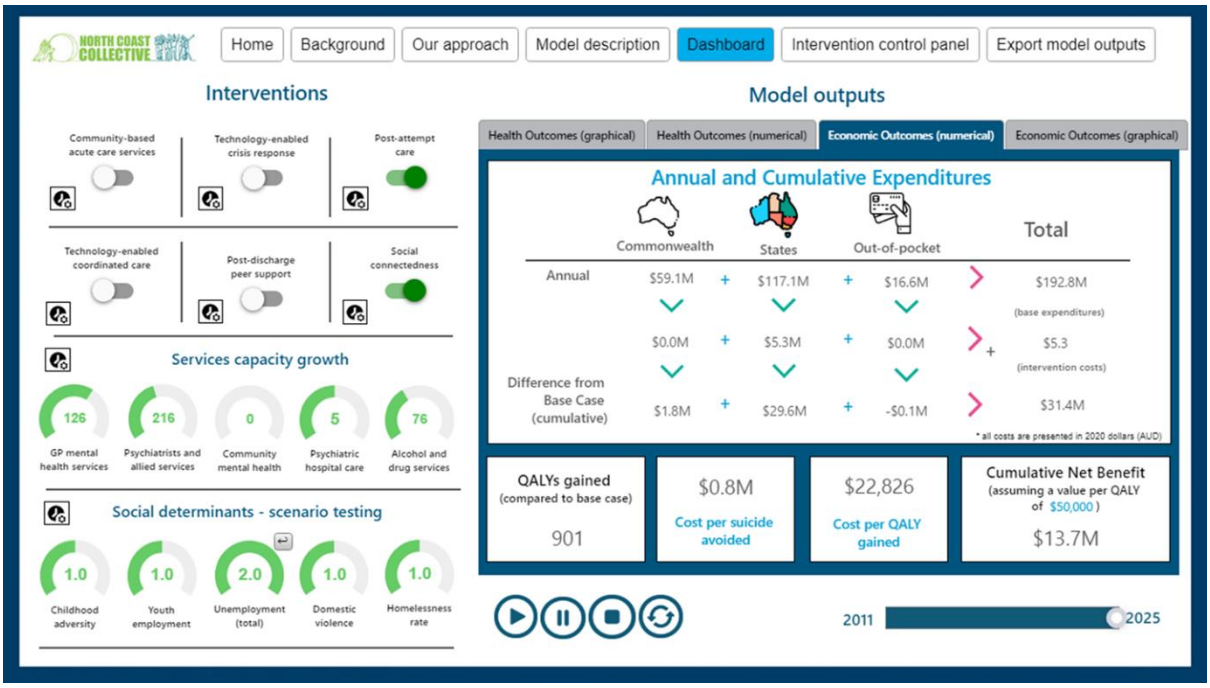

Supplementary figure 2 Example dashboard for economic outcomes

There then may be further sections to represent societal perspective and specific pages to show how cost and outcome change over time. Uncertainty analysis can be shown separately.

Stage 8: Using the economic outcomes to help develop business cases

The economic outputs from the model can be used to support local decision makers to prepare business cases regarding investment in intervention(s). As part of the participatory process, there will be a separate ‘user guide’ for local service staff who are not economists or modellers to interact with the model, with a step-by-step process to generate model outputs, including economic outputs. The purpose of this section of the SOP is for the economist(s) involved in the research project to generate the key economic information that can be included to support business cases. The focus here is summarising the information required to be entered into business cases, with recommended format of associated tables.

There are two main purposes of business cases. The first is to influence decision-making within the organization (e.g. PHN) itself with respect to how to allocate and/or reallocate current resources with the aim of maximising an outcome(s) of interest. The second is external to the organization with respect to bidding for additional resources, such as the Department of Health (Commonwealth or State) and Treasury, if the amount required is in-excess of $10 million, as further discussed below.

A. Business cases for discussion *__within__* **the** *service organization to allocate current budgets*

The economist should ensure that the model outputs can produce the following summary table and economic metrics:

Supplementary table 1: Economic valuation from a health sector perspective

Intervention(s)	$ Investment	$ service costs	QALYs	Cost per QALY	Overall: $ Health net benefit
1					
2					
N					
1+2+N					

**N is other interventions, $ is change relative to BAU, Net benefit is overall impact on health sector*

Each row in the table would be the cumulative values over the chosen model run time (e.g. 5, 10, 20 years). If annual amounts are required, in additional to cumulative, these can be downloaded from the model directly and exported to excel, for example. Discounted values should be used in keeping with best practice, notwithstanding a sensitivity analysis as described above where the discount rate can be varied, including undiscounted rates.

The table then provides the key metrics, including the investment required in a specific intervention to enable the organisation to understand affordability. The impact on other service activities and the associated costs is also estimated to understand the flow-on impacts (e.g. GPs, hospitals). Quality-adjusted life years generated (QALYs) summaries the impacts on extending life expectancy and (health-related) quality of life as a measure of overall health benefit. The $ cost per QALY is the investment cost (plus/minus) the change in service costs divided by the QALYs. A value of under $50,000 is a rule of thumb to indicate cost effectiveness. Finally, net benefit is a measure of overall financial value, where QALYs are multiplied by $50,000 and summed with net costs, where positive values indicate a positive benefit for the investment made. Note, the particular metrics may vary if stakeholders feedback preferences for particular information. The economist should be responsive while also adhering to methodological guidelines. To reiterate, the economist role is to equip the model to undertake a range of analysis and generate different metrics so the SD models can be used flexibility.

The organisation using the model can then draw upon this information to understand the costs and benefits of different interventions and combinations. When combining interventions (N) there is likely to be non-additive impacts (synergetic or antagonistic impacts) which is the key strength of the SD modelling and economic approach. The organisation can then choose the time frame of the model projections appropriate for particular planning horizons.

Overall, this summary information is intended to support organisations to improve population health subject to resource constraints and help communicate to communities (where necessary) the rationale for particular resource allocation decisions made (e.g. intervention combination ‘x’ produces greater health gains than combination ‘y’).

B. Business cases to bid to external agencies for additional funding

Service organisations will also be able use the economic outputs to make the case for additional funding to expand existing services and/or invest in new interventions. This may involve bids to, for example the Department of Health (State, Commonwealth). Note, Treasury departments now require any funding bids that reach $10 million or above be accompanied by a formal business case and where the supporting economic information includes, but extends beyond, the health sector to consider multi-sector impact and wider economic impacts, if relevant. Potential wider impacts may include the impact on non-health sectors (e.g. social services) should the model scope simulate these impacts which will be decided in the participatory workshops. The impact on carers can also be included, extending to the monetized quality of life impacts, and lost productivity.

Supplementary table 2: Economic valuation from a societal perspective

Intervention(s)	Investment	$ Health net benefit	$ Carers	$ Productivity	Overall: $ Society net benefit
1					
2					
N					
1+2					

** Health sector net benefit taken from table above. Net benefit for society is health sector plus other impacts combined.*

An additional metric is also required, the Benefit Cost Ratio (BCR). Using the information above, this is [$Net benefit (society) - $ investment] / $ investment. A ratio above 1 indicates the investment is a net gain to society. A ratio less than 1 indicates that resources should not be invested and reallocated to other uses. In situations, where an organisation received an increase in budget unspecified to particular programs, this can be optimally allocated using the constrained optimisation approach, as described in the protocol.

It is important to note that this economic information are inputs into a business cases which also needs to contain separate financial analysis and governance arrangements. Further information regarding the key elements of a business cases can be accessed: https://www.treasury.nsw.gov.au/information-public-entities/business-cases

Stage 9: Communicating how the model can be implemented going forward in dynamic economic priority setting exercises

Workshop 3 will illustrate how stakeholders can use and interact with the model, in general, use the dashboard and test out service interventions (both individually and in combination), demonstrating potential impacts relative to business-as-usual. The emphasis of this process to increase user confidence in switching interventions on/off and varying implementation timing, and support users to interpret model outputs. When presenting at Workshop 3, the economist should communicate that further research and analyses post-Workshop is possible to inform economic priority setting, as outlined below. The presentation at the workshop should outline that this would be led by the economist(s) in partnership with both the technical and participatory modelling teams. The intention is to provide a placeholder for further applied research. It would also serve to help promote the sustained use of the model going forward within local governance structures and informing real-world decisions.

Participatory approach to Program Budgeting Marginal Analysis (PBMA):

There are existing priority setting exercises, such as Program Budgeting Marginal Analysis (PBMA). https://resource-allocation.biomedcentral.com/articles/10.1186/1478-7547-2-3 These typically involve a senior management team responsible for the allocation of budgets between programmes and a research team to facilitate the prioritisation process. There may be an opportunity to enhance these approaches by using the SD models and associated economic analysis.

Briefly, the presentation at Workshop 3 should first acknowledge that when setting priorities economic metrics are one key input alongside other concerns such as pragmatic constraints (e.g. contract commitments) and ethical considerations (e.g. equity judgements). The first task of the priority setting exercise would be to define the scope, including budget available and candidate programmes/interventions for investment and disinvestment. The modelling can then be used to help identify the optimal combinations, as follows:

Dynamic Multi-Criteria Decision Analysis (MCDA)

As part of the PBMA exercise the constrained optimisation algorithm can be employed to identify the best value portfolio of interventions to allocate budgets towards. The algorithm can be adjusted to include other concerns as appropriate and feasible. For example, the Multi-Criteria Decision Analysis (MCDA) can consider a range of potential policy objectives, such as different outcomes and equity considerations such as giving more value to outcomes achieve in certain population groups, if baseline inequities for instance https://resource-allocation.biomedcentral.com/articles/10.1186/s12962-018-

0118-7. If needed, an overall weighted outcome or metric can be developed by eliciting stakeholder preferences regarding which outcomes have greater importance related to others. These preferences serve as the weights to combine different objectives into an overall metric of value.

The SD model can be used to embed the weighted outcome(s), alongside intervention options, time frame to desired impact, and budget available. In addition, when identifying optimal interventions it will be important to estimate the potential consequences of expanding one service (e.g. GP screening) on other services elsewhere in the system (e.g. allied health) to ensure that if an interventions is implemented that other parts of the system are sufficiently prepared for additional population flows entering services and so resourced adequately.

The economic analysis and modelling would automatically develop the optimal portfolio, however, stakeholders themselves would be invited to choose the outcome to be optimised. This can include changing the valuation metric, which involved normative or ethical judgements, such as whether we optimised the return on investment (i.e. only invest to save), or optimise societal benefit (i.e. investing to maximise societal outcomes). Further, this exercise can be updated periodically should priority setting processes become part of governance processes going forward.

In summary, the economic priority setting exercises would be rooted in stakeholder co-design, developed in a transparent way to represent local preferences to tailor investment portfolios to local needs and impacts. There may be different proposed interventions for each of the eight study sites. The protocol and SOP has intended to describe the generic processes that will be undertaken across all sites to develop local solutions for local needs and create maximise value for the resources available to improve mental health and wider economic outcomes.
